# A Manual of Diseases of the Throat and Nose

**Published:** 1884-11

**Authors:** 


A MANUAL OF DISEASES OF THE THROAT AND NOSE, including the Pharynx, Larynx, Trachea, Œsophagus, Nose and Naso Pharynx. By Morell Mackenzie, M. D., London, Consulting Physician to the Hospital for Diseases of the Throat; Lecturer on Diseases of the Throat at the London Hospital, Medical College, and Corresponding Member of the Imperial Royal Society of Physicians of Vienna. Vol. II. New York, Wm. Wood & Co. 1884.

In the medical world, McKenzie is regarded as the best authority on diseases of the throat. The last of his writings, under the above title, is not only instructive, but exceedingly entertaining reading matter. The profession at large knows very little about throat and nasal diseases in general, considering them obscure, difficult to treat, and in chronic cases often regarding them incurable, or at best terminating very unsatisfactorily.

The author makes plain and easily understood what at first sight appears obscure and difficult, and both for the general practitioner and specialist throws much light upon hitherto faintly comprehended subjects.

The chapter on stricture of the œsophagus is worthy of careful study by every physician. It is fortunately not a very common disease, but occasionally such a case falls into the hands of every doctor. The knowledge that some of these patients can be cured is most comforting to him who has formerly considered them incurable.

Equally interesting is the chapter on laryngeal phthisis, in the first volume. While by means of medicines we are very little more capable than formerly of bringing this painfully terrible disease to a favorable ending, still it is of incalculable advantage to be able to recognize the disease in its very incipiency and sound the note of warning to the patient and his friends. The tubercular deposits or small ulcers in the region of the vocal cords or upon the epiglottis, can be readily seen by one who is at all conversant with the use of the laryngoscope.

The volume is a valuable addition to the literature and therapeutics of throat and nasal diseases. A. W. C.

